# Evaluating Economic Growth, Industrial Structure, and Water Quality of the Xiangjiang River Basin in China Based on a Spatial Econometric Approach

**DOI:** 10.3390/ijerph15102095

**Published:** 2018-09-25

**Authors:** Xiaohong Chen, Guodong Yi, Jia Liu, Xiang Liu, Yang Chen

**Affiliations:** 1 School of Business, Central South University, Changsha 410083, China; cxhcsu@126.com (X.C.); 141601020@csu.edu.cn (G.Y.); 161601012@csu.edu.cn (Y.C.); 2 Institute of Big Data and Internet Innovation, Hunan University of Commerce, Changsha 410205, China; 3 School of Information and Safety Engineering, Zhongnan University of Economics and Law, Wuhan 430073, China; 4 Institute of Finance, Guangzhou University, Guangzhou 510006, China

**Keywords:** economic growth, water quality, spatial effects, industrial structure

## Abstract

This research utilizes the environmental Kuznets curve to demonstrate the interrelationship between economic growth, industrial structure, and water quality of the Xiangjiang river basin in China by employing spatial panel data models. First, it obtains two variables (namely, COD_Mn_, which represents the chemical oxygen demand of using KMnO_4_ as chemical oxidant, and NH_3_-N, which represents the ammonia nitrogen content index of wastewater) by pretreating the data of 42 environmental monitoring stations in the Xiangjiang river basin from 2005 to 2015. Afterward, Moran’s I index is adopted to analyze the spatial autocorrelation of COD_Mn_ and NH_3_-N concentration. Then, a comparative analysis of the nonspatial panel model and spatial panel model is conducted. Finally, this research estimates the intermediate effect of the industrial structure of the Xiangjiang river basin in China. The results show that spatial autocorrelation exists in pollutant concentration and the relationship between economic growth and pollutant concentration shapes as an inverted-N trajectory. Moreover, the turn points of the environmental Kuznets curve for COD_Mn_ are RMB 83,001 and RMB 108,583 per capita GDP. In contrast, the turn points for NH_3_-N are RMB 50,980 and RMB 188,931 per capita GDP. Additionally, the environmental Kuznets curve for COD_Mn_ can be explained by industrial structure adjustment, while that for NH_3_-N cannot. As a consequence, the research suggests that the effect of various pollutants should be taken into account while making industrial policies.

## 1. Introduction

Water is an indispensable substance for human life, economic development, and social progress. With the rapid development of the economy and frequent human activities, the demand for water is increasing, which gives rise to pressure on ecosystems, and especially water ecosystems. At present, a water crisis that includes water shortages, pollution, and quality deterioration is increasingly prominent and has become the main bottleneck in restricting sustainable development of human economy and society [1].

Xiangjiang River is the largest river in the Hunan Province, China. With dense towns, a large population, and concentrated industry, the river basin hosts 60% of the population and 81% of the total industrial output value of Hunan Province, which makes it the main economic belt with the densest population and fastest urbanization development in Hunan [2]. However, the rapid development of the economy and constant discharge of industrial, domestic, and agricultural wastewater in recent years have resulted in the degeneration of the Xiangjiang River Basin’s ecological function. Even worse, the discharge of pollutants in some river sections has exceeded the maximum capacity that a natural water environment can stand. The severe exceeding of ammonia nitrogen, total phosphorus, and chemical oxygen demand in Xiangjiang River Basin has led to horrible river pollution in the Hunan Province [3,4]. In view of this, pollution control in the Xiangjiang river basin is highly valued by the state and local governments of China. In July 2009, the Chinese government launched a special treatment project for heavy-metal pollution in the Xiangjiang basin, investing 595 billion yuan in total. In March 2011, the State Council of China approved the implementation plan for the treatment of heavy-metal pollution in the Xiangjiang basin, which has become the first pilot project of regional heavy-metal pollution control by the State Council of China. The main flow area in the Xiangjiang basin, Chang-Zhu-Tan city group, became the pilot area constructing a two-oriented society in 2007, which is a major policy measure to explore ways for conserving resources and protecting the environment without damaging economic growth in China. A series of policies to protect the Xiangjiang river were also implemented here, such as emission trading and ecological compensation institutions.

Economic growth has had significant impact on changes of environmental quality. Consumption of resources and pollutant discharge caused by economic growth inevitably bring about environmental degeneration. Meanwhile, economic growth could reduce pollutant discharge and improve the environment by advancing technology, improving the economic scale, as well as increase revenue. The debate over the role that economic growth plays in determining environmental quality has been rapidly gaining importance [5,6]. Starting with the influential studies of Grossman and Krueger [7], and Selden and Song [8], a number of studies on the hypothesis of an Environmental Kuznets Curve (EKC) have investigated this relationship for various pollutants, regions, and time periods [2,9].

The EKC hypothesis depicts a long-term relationship between economic growth and environmental quality [10]. According to this view, the link between environmental quality and economic levels varies with the economic development stage. As a consequence, in the early stages of the development process, economic growth could lead to environmental degradation in the early stages of the development process, but once economic development exceeds a certain threshold, further increase in per capita income would help reduce pollution emissions and improve environmental quality. In addition, according to Panayotou [11], a high development level would lead to better regulations, greener technologies, and higher expenditures to protect the environment. At present, a large number of studies on EKC at home and abroad have selected many specific pollutant indicators such as atmosphere, water bodies, and solid waste in different regions for analysis and verification. In a meta-analysis of 121 usable observations gathered from a set of 25 studies, Cavlovic et al. [12] found that environmental indicators are divided into 11 categories, namely, toxic emissions, urban air quality, deforestation, heavy particulate, urban quality, water quality/pollution, heavy metals, SO_2_, combustion byproducts, hazardous waste, and CO_2_.

It is possible that, in analyzing situations of different countries or different periods of a single country, researchers have drawn different conclusions because of the use of different regression equations, pollution indicators, or control variables. In other words, the relationship presented multiple and mixed types [13,14]. Some scholars found a traditional inverted U-shaped trend [15,16,17], yet others found that the turning point does not exist, but shows a linear relationship [18]. Moreover, some scholars found an N-shaped or inverted U-shaped relationship [19,20], while others, like Moomaw and Unruh (1997) [21], found that the relationship varied greatly in different countries [22,23,24] or even that there was no significant relationship between economic growth and environmental quality [25]. In addition, there was also evidence that testing results depended on specific econometric models [26,27]. In summary, the EKC has shown various forms, such as U-shaped, N-shaped, and linear. In other words, various environmental indicators of different regions do not follow a specific evolutionary trajectory.

In existing empirical studies on EKC, model analysis is usually performed based on cross-sectional data from different geographic regions or administrative divisions. A general assumption is that pollution emissions between regions are uncorrelated. That is to say, the economic growth of a region only affects the environmental quality of itself, rather than the surrounding environment, which is obviously inconsistent with reality. It should be taken into consideration that environmental pollution may exist in spatial autocorrelation or spatial dependence [28]. On the one hand, almost all spatial data might have spatial or spatial autocorrelation characteristics [29], and pollution-emission data may be no exception. On the other hand, there are strong regional clusters of pollution-source distribution, energy-consumption structure, and pollution-control investment capacity in different regions, which also makes pollution discharge have a certain spatial correlation [30]. If the effects of this spatial correlation are ignored, the estimation of the EKC model might have a bias or a parametric test that produces errors. Therefore, spatial metric analysis methods should be used when dealing with EKC-related regional data [31,32,33].

However, there are not many literatures using spatial econometric models to study EKC, or especially dealing with the watershed environmental problem. Su et al. [34] employed spatial regression to analyze the determinants of dissolved oxygen (DO) and nutrients in the Qiantang River, China. Chang [35] examined spatial patterns of water-quality trends for 118 sites in the Han River basin of South Korea. Wang et al. [36] employed statistical tools, the GIS technique, and the EKC method to analyze the spatiotemporal relationship of riverwater quality with the economic growth in the Jiulong River Basin of the Western Taiwan Strait Economic Zone. Yang et al. [37] utilized spatial regression to evaluate the impact of watershed characteristics on stream NO_3_NO_2_-N concentration in the Cedar River Watershed, Iowa. In a meta-analysis of 255 EKC studies published between 1995 and 2010 [38], only 35 studies used water-quality indicators as the dependent variable in comparison to 214 studies that used air, climate, and energy indicators. Of the few studies that focused on water pollution, only 10 have performed at a lake and watershed level. Paolo et al. [39] examined the nexus between economic growth and water usage using a country’s water footprint as an indicator of water impact and found an N-shaped curve for the relationship between water footprint per capita and GNI per capita, and grey water footprint per capita and GNI per capita.

In view of this, this research utilizes the environmental Kuznets curve to demonstrate the interrelationship between economic growth, industrial structure, and water quality of the Xiangjiang river basin in China by establishing spatial panel data models. First, it obtains two variables (COD_Mn_ and NH_3_-N) by pretreating the data of 42 environmental monitoring stations in the Xiangjiang river basin from 2005 to 2015. Afterward, Moran’s I index is adopted to analyze the spatial autocorrelation of COD_Mn_ and NH_3_-N concentration. Then, a comparison between the nonspatial panel model and spatial panel model is conducted and the mediating effect of the industrial structure is evaluated. The remainder of this paper is organized as follows: the second part introduces the EKC model and the spatial econometric model with an EKC hypothesis; the third part describes the research site, data processing, and the correlation and multicollinearity of the variables; the fourth part is the empirical results and discussion; the fifth part draws conclusions and policy recommendations.

We contribute to the emerging literature in several ways: first, for the research method, the inverted N-shaped space panel EKC model is adopted; second, regarding the research content, the EKC formation mechanism of a watershed is analyzed from the perspective of industrial-structure adjustment; and third, for the research data, nonpublic pollutant-concentration data collected by the government, rather than commonly used emission data, are used.

## 2. Materials

### 2.1. Study Site

**Figure 1:**
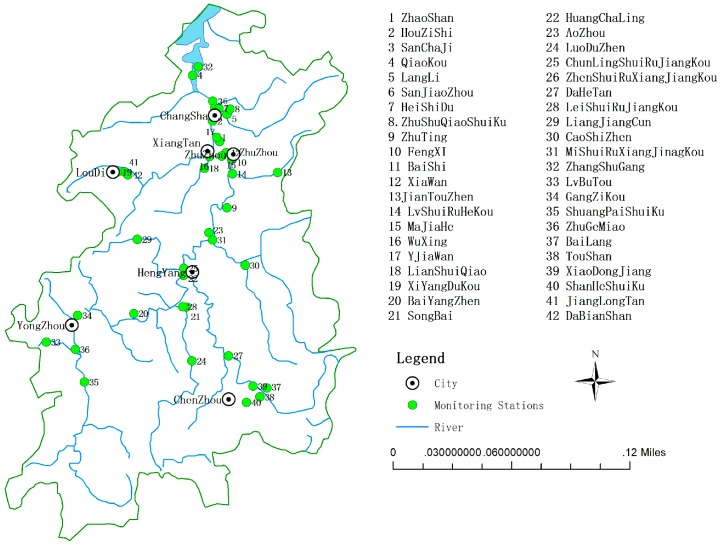
Location of environmental monitoring stations in the Xiangjiang river basin.

The Xiangjiang River, which traverses from its source in Xin’an of the Guangxi Province to its confluence with the Yangtze River in Dongting Lake, is one of the major and important rivers in Hunan Province, southern China. The location of each environmental monitoring station in Xiangjiang River Basin is shown in Figure 1. The river has a length of approximately 856 km (with 660 km in the Hunan Province), a catchment area of approximately 94,660 km^2^ [40], and it covers eight cities, namely, Yongzhou, Chenzhou, Hengyang, Loudi, Zhuzhou, Xiangtan, Changsha, and Yueyang. The water of the Xiangjiang River is used for irrigation, and domestic and industrial purposes. In the Hunan Province, there are abundant reserves of nonferrous metals. Most of the ores used for mining, mineral processing, and the smelting of nonferrous and rare metals are found in the middle and lower reaches of the Xiangjiang River, while effluents from these intensive mining and industrial activities are heavily loaded into the river [41]. Moreover, many large cities, such as Changsha, Xiangtan, and Zhuzhou, are located in the middle and lower reaches of the Xiangjiang River. With the development of industrial production and the enlargement of cities, the middle and lower reaches of the Xiangjiang River have been seriously polluted in recent years [42]. Various heavy industries operate in the Xiangjiang river basin, including chemical, dyeing, electroplating, and food plants. In the last eight years, this area has nearly doubled its GDP, increasing from about 190 billion dollars in 1996 to 360 billion dollars in 2003. This basin exemplifies some direct water-pollution problems, and many typical water-pollution problems that occur during the process of urbanization in developing countries. The case study of the Xiangjiang River, therefore, provides a good opportunity to examine the spatial determinants of water pollution.

### 2.2. Data and Processing

Existing research indicates that water quality is mainly affected by point-source pollution that is caused by industrial wastewater and urban/domestic sewage and nonpoint-source pollution that is caused by agricultural activities, soil erosion, and solid waste [43,44,45]. As GDP represents the level of social and economic development of a place, in general, the higher the GDP is, the more frequent corresponding economic activities are, and the more obvious the environmental impact is. With the development of economy and society and the constant improvement of urbanization, the contradiction between population increase and a serious shortage of water resources is becoming increasingly conspicuous. Population density can directly reflect the social production activities of a region. Higher population density leads to a higher demand for water and greater pollutant emissions. Natural precipitation also brings the pollutants of the surrounding areas into the river and further pollutes the water.

With a dense population, developed economy, and high-level urbanization, the Xiangjiang river basin area enjoys the rapid development of agriculture and forestry, a large number of enterprises, and abundant rainfall. The main pollution of the area is derived from industrial pollution, domestic wastewater pollution, and agricultural pollution, among which heavy-metal pollution is particularly serious [46,47]. Pollutants mainly include COD_Mn_, NH_3_-N, mercury, arsenic, and hexavalent chromium. In this paper, we focus on the eutrophication of the Xiangjiang river basin that is caused by organic pollution and nutrients. The quality of the measured data of total phosphorus is extremely poor, many of which are lower than the minimum detection index of 0.01. Consequently, we selected COD_Mn_ and NH_3_-N as the water-quality indicators of Xiangjiang river basin; and took per capita GDP (pergdp), primary industry (primary), industry (indus), population (pop), urbanization level (ur), and precipitation as control variables.

In this paper, we extracted water-quality data from the 42 environmental monitoring stations in the Xiangjiang river basin. Then, we matched the 42 monitoring stations with corresponding counties according to map coordinates. It should be noted that (1) there are more than one monitoring sites in some areas, for example, Bailang, Toushan, and Xiao Dongjiang are nestled in Zixing City, so we regarded the average data in the above three places as the environmental quality data in Zixing City; (2) several monitoring stations measure more than one district. For instance, the Lilang monitoring station supervises the Yuhua District and Changsha County. Thus, we merged the above two places into one area in our article and took the sum data as the research object. Based on this, 26 observations were selected.

Additionally, the data related to the economic income and social development of the Xiangjiang river basin were obtained from the “2005–2015 Statistical Yearbook of Hunan Province”, among which GDP (pergdp) was treated at a constant price based on the 2005 price index. The unit of population (pop) density was “Ten thousand people per square kilometer”, primary industry (primary) refers to the proportion of the added value of the primary industry to the GDP, and industry (indus) refers to the proportion of industrial added value to the GDP.

### 2.3. Correlations and Multicollinearity of Variables

The monitoring results revealed that the average concentration of COD_Mn_ ranks the highest from 2005 to 2015 of all the pollutants in the Xiangjiang River Basin. Table 1 shows that the maximum value was 8.27 mg/L, while the minimum was 0.25 mg/L. In addition, average concentration of NH_3_-N was nearly 0.64 mg/L, which is also at a relatively high level. Specifically, the concentration of COD_Mn_ and NH_3_-N in the Liuyang and Zhenshui rivers (the tributaries of Xiangjiang and the corresponding monitoring stations are SanJiaoZhou, HeShiDu, and ZhenShuiRuXiangJiangKou) were higher on the whole, while reaching an average of 4.65 mg/L and 1.89 mg/L, respectively. The main reason is that a large number of industrial and papermaking enterprises are distributed on both banks of the Liuyang and Zhenshui rivers, but sewage discharge has not been effectively controlled. However, the concentrations of the two pollutants showed a trend of rising first and then decreasing because of the comprehensive remediation of environmental pollution in the basin. The main measures include the regulation of livestock and poultry-farming pollution, elimination and renovation of industrial pollution sources, and strengthening centralized and harmless disposal of domestic sewage and domestic waste in towns and townships.

**Figure 2:**
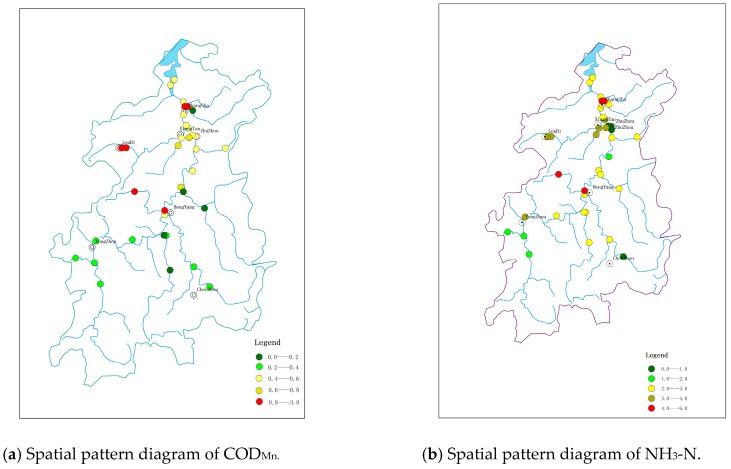
Spatial pattern diagram of water-environment quality.

Data-analysis results demonstrate that the concentration of major pollutants in each section of the Xiangjiang river basin is gradually increasing from upstream to downstream. Figure 2 reveals the pollution of major cities along the Xiangjiang river basin. The darker color indicates the higher concentration of contaminants. It can be seen from the figure that the concentrations of COD_Mn_ and NH_3_-N of Changsha, Loudi, and Xiangtan in the lower reaches of the basin are significantly higher than those of Chenzhou and Yongzhou in the upstream part.

Table 2 shows the variable correlation coefficient matrix. It can be seen that the correlation coefficients of most variables are low or moderate. Most variables have strong and significant correlation, while the correlation between COD_Mn_ (NH_3_-N) concentration and primary (precipitation) is rather weak and insignificant. In addition, there is a certain correlation between every two control variables. To further test multicollinearity, a Variance Inflation Factor (VIF) test was used. The VIF test showed that the biggest value is 9.32, which indicates that multicollinearity might exist. We also found that the variable (ur) had no significant effect on the COD_Mn_ (NH_3_-N) coefficient. When the (ur) was deleted, the biggest value of VIF became 4.93, indicating that there was no multicollinearity between the variables [2,48].

## 3. Methodology

Spatial regression was employed to analyze the relationship between water-pollution indicators and economic growth considering the spatial correlation of water pollution. Spatial regression models typically include three categories, which are spatial lag (SAR, Equation (1)), spatial error (SEM, Equation (2)) and spatial Durbin mode (SDM, Equation (3)). Spatial lag regression is expressed as follows:

(1) $$y_{it}=\lambda \sum w_{ij}y_{it}+\beta x_{it}+\alpha_{i}+\gamma_{t}+\varepsilon_{it}\;$$

where $y_{it}$ is the water-pollution indicator of monitoring station *i* in year *t*. $x_{it}$ is the GDP of area *i* in year *t* indicating the level of economic growth. $Z_{it}$ is the set of other explanatory variables, including population density, rate of urbanization, and industrial structure. $\alpha_{i}$ are unobserved country-specific effects; $\gamma_{t}$ is the time-specific effect; $\varepsilon_{it}$ is the normally distributed error term; λ is the spatial autoregressive coefficient; and $\left(w_{ij}\right)$ is the spatial weight matrix that describes the relationship between every two sites.

Spatial error regression is given as:

(2) $$\begin{array}{l} y_{it}=\beta x_{it}+\alpha_{i}+\sigma_{it}\\ \sigma_{it}=\lambda {\sum w}_{ij}\sigma_{it}+\varepsilon_{it} \end{array}$$

where $\sigma_{it}$ denotes the spatially autocorrelated error term. Term λ reflects the spatial autocorrelation coefficient of the error term. The other parameters are the same as the above mentioned.

Some spatially autocorrelated variables in the spatial-error model were omitted in the regression, while the model consequently generated a spatially autocorrelated error. The spatial lag model assumes that, besides explanatory variables, the water-quality variable in one site is affected by the spatially weighted concentrations in its neighborhood. The SAR and SEM models both consider county interactions. However, when studying the spatial difference of river pollutants, water flow direction from upstream to downstream directly leads to unidirectional (from upstream to downstream) spatial influence. At the same time, a spatial relationship occurs when the dependent variable can be predicted as a function of spatially lagged values of the independent variables. Therefore, the two spatial-interaction situations need to be considered simultaneously. The spatial Durbin model specification provides a function containing spatially lagged values, both of the dependent variable and the independent variables, which is specified as:

(3) $$y_{it}=\lambda \sum w_{1ij}y_{it}+\beta x_{it}+\lambda {\sum w}_{2ij}X_{it}+\alpha_{i}+\delta_{t}+\varepsilon_{it}\;$$

$w_{1ij}$ is a unidirectional contiguity matrix, namely $w_{1ij}$ = 1 if *j* is the nearest upstream region of *i*, otherwise $w_{1ij}$ = 0. $w_{2ij}$ is a threshold distance and the spatial matrix in which the element $w_{2ij}$ is calculated using the square of the reciprocal of the geographic distance between counties *i* and *j*. Distance-based weights are defined as follows:

(4) $$\begin{array}{l} w_{2ij}=0,\;\mathrm{if}\;i=j\\ w_{2ij}=1/d_{ij}^{2},\;\mathrm{if}\;d_{ij}\leq 77\\ w_{2ij}=0,\;\mathrm{if}\;d_{ij}>77 \end{array}$$

where $d_{ij}$ is the distance in kilometers between counties *i* and *j.* The 77 km distance was the cut-off parameter above which interactions were assumed to be negligible. This distance was chosen so that each county interacted with at least one other county. This cut-off parameter is important since there must be a limit to the range of spatial dependence allowed by the spatial-weight matrix [49,50,51]. This is due to the asymptotical features required to obtain consistent estimates for the model parameters. We use the inverse-squared distance in order to reflect gravity relation. The two matrices were both row-standardized so that each row summed to one and the coefficients that emerged from the subsequent regression could be readily interpreted by virtue. Under the definitions of $w_{1ij}$ and $w_{2ij}$, the spatial Durbin model took account of the two interaction cases simultaneously.

Due to the introduction of a spatial-weight matrix, spatial econometric models arouse an endogenous variable problem. If spatial econometric models were still estimated by ordinary least squares (OLS), estimated coefficients would be biased. Therefore, spatial panel models are generally estimated with the maximum likelihood method [52]. As each county has characteristics that do not change or change very little over time, such as unobservable geographic characteristics and resources endowment, this can lead to parameter homogeneity in each spatial unit. In this paper, we primarily focused on the fixed effects of the estimation procedure. The time period affected control for time-specific shocks that affect economic factors such as economic crises, oil shocks, and national environmental policy, which leads the coefficients to vary in each time unit. For completeness, we also estimated the random-effect model and used the Hausman diagnostic test to determine which model provided a better fit for the data. Two models were analyzed, while the Robust Lagrange Multiplier (LM) tests and their robustness (Robust-LM) were used to determine the form of spatial relationships. To verify if the spatial panel data models offered a more appropriate specification, we tested which spatial panel data model was the best-fitting for the data with the Wald and LR tests. We used the null hypotheses (H0: γ = 0) of the Wald test to examine whether the SDM model could be simplified to the SAR model. We also explored the null hypothesis (H0: γ+βλ = 0) of the LR test to determine whether the SDM model could be simplified to the SAR model or SEM model. If both of the null hypotheses were rejected, the spatial Durbin model provided the best fitting.

## 4. Empirical Results and Discussion

### 4.1. Spatial Autocorrelation for COD_Mn_ and NH_3_-N

This part uses Moran index analysis to comprehensively evaluate the spatial autocorrelation of COD_Mn_ and NH_3_-N. The Moran Index was introduced by Australian statistician Patrick Alfred Pierce Moran in 1950. The range of Moran’s I values concentrates in the interval (−1, 1), where 1 and −1, respectively, denote the strongest positive spatial autocorrelation and strongest negative spatial autocorrelation [53]. Table 3 suggests that Moran’s I index values of the COD_Mn_ and NH_3_-N in the Xiangjiang River from 2005 to 2015 were all positive and in the range of (0, 1), which indicates that there exists spatial autocorrelation between the two types of pollutants. Especially for NH_3_-N, its Moran index reached the highest—0.871 in 2015. Therefore, the river COD_Mn_ and NH_3_-N patterns depended both on neighboring patterns and on a set of local independent parameters. All these results demonstrate the need to incorporate spatial analysis when interpreting the spatial determinants of COD_Mn_ and NH_3_-N.

### 4.2. Empirical Estimation and Results

A suitable model is a prerequisite for reliable empirical results. In order to detect which model best fits the data, this paper first conducts a nonspatial panel model analysis, then examines whether there exists spatial correlation among spatial units by the classic LM tests and the Robust LM tests. As shown in Table 4 and Table 5 referring to the results of classical LM tests on COD_Mn_, the null hypothesis of no spatially lagged dependent variable and the null hypothesis of no spatially autocorrelated error term were respectively rejected at 10% and 5% significance level. In the robust LM tests, both of the hypotheses were not rejected when the OLS is included. While referring to the results of classical LM tests on the NH_3_-N, both of the hypotheses were strongly rejected at a 1% significance level, while both of the hypotheses were rejected for the OLS in the robust LM tests. These results show that spatial panel models are better than nonspatial interaction effects of traditional mixed panel data models. In terms of a model-fitting effect, the SDM model had the most significant regression coefficient compared with the SAR or SEM model. To further judge the fitting effect of the SDM model, we estimated the spatial Durbin model and then performed the Wald and LR tests. According to the results of the Wald and LR tests on COD_Mn_ and NH_3_-N, both of the null hypotheses were rejected at the 1% significance level. Furthermore, we conducted the Hausman test, and the result of COD_Mn_ and NH_3_-N were respectively rejected at 5% and 1% significance level. These results imply that the SDM model is more appropriate than the SAR or SEM model in reflecting the impact of spatial autocorrelation on regression results. Therefore, we adopt the SDM model with fixed effects to study the spatial effect of economic development on water quality in the river basin.

**Figure 3:**
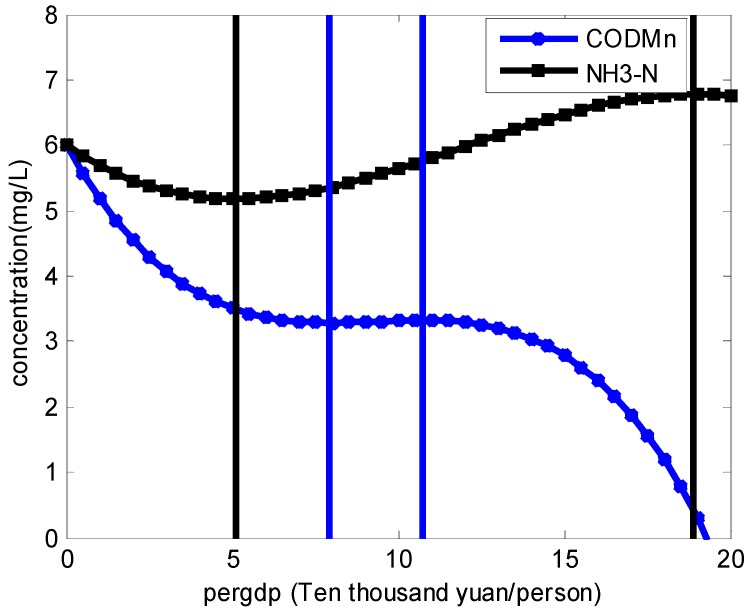
Environmental Kuznets Curve (EKC) of COD_Mn_ and NH_3_-N. Notes: Blue curve denotes COD_Mn_ and the black denotes NH_3_-N. Overall concentration of NH_3_-N is actually lower than that of COD_Mn_, the curve of it is adjusted upwards for the aesthetics of the figure.

This shows that, for COD_Mn_ and NH_3_-N, estimated coefficients of the cubic polynomial of per capita GDP (Pergdp3) are highly significant, which presents that the relationship between economic growth and the concentration of these two pollutants cannot validate the traditional environmental Kuznets curve hypothesis. However, it shows a back-N style, as displayed in Figure 3. The first turning point of the environmental Kuznets curve for COD_Mn_ is at approximately RMB 83,001; the second turning point is at approximately RMB 108,583. By contrast, for NH_3_-N, the first turning point is at approximately RMB 50,980; the second turning point is at approximately RMB 188,931.

The EKC of other watersheds are different, for example, Jiang (2014) [46] studied the EKC of the Qiantang river basin districts in Hangzhou, Jinhua, and Quzhou from 2005 to 2012; the results showed that the EKC was U-shaped, inverted U-shaped, and N-shaped. Moreover, the EKC of the Changjiang river basin and Taihu river basin was U-shaped; of the Haihe river basin, and Huanghe river basin was S-shaped; and of the Huaihe river basin and Liaohe river basin was tilted-S-shaped. Compared with these basins, the EKC of the Xiangjiang river basin was inverted N-shaped. The reason may be due to a series of targeted environmental protection measures being implemented in the Xiangjiang River Basin, especially since 2005, such as the Comprehensive Management Plan of Ecological Environment in Xiangjiang River Basin of Hunan Province (2009), the Hunan Xiangjiang Protection Regulations (2012), and the Regulations on Prevention and Control of Water Pollution in Xiangjiang River Basin, Changsha City (2016). These laws and regulations regulate the economic activities of the river basin in specific aspects to improve environmental quality.

Taking the 2016 data as an example, for the environmental Kuznets curve of the pollutant NH_3_-N, the per capita real GDP of 11 districts or counties of the Xiangjiang river basin are in the upward phase of the inverted “N” curve, namely, Lusong District, Shifeng District, Yuelu District, Kaifu District, Yuhua District/Changsha County, Furong District, Tianxin District, Wangcheng District, Xiangtan City, Louxing District, and Zixing City. As for the environmental Kuznets curve of the pollutant COD_Mn_, 10 districts or counties are in the upward phase, namely, Lusong District, Shifeng District, Yuelu District, Kaifu District, Yuhua District, Changsha County, Tianxin District, Wangcheng District, Xiangtan City, and Zixing City.

Since the diagnostic results above have suggested that the SDM with fixed effects is the best fit, we limited the interpretation of coefficient estimates to it. As shown in Table 4, focusing on the estimated coefficient of primary industry (primary), industry (indus), and population (pop), the elastic coefficients were −6.726415, −2.326853 and −2.405921, respectively. By contrast, the results described in Table 5 are 1.028606, 0.6328895, and −4.974618, which indicates that primary industry (primary) and industry (indus) have a negative effect on increasing COD_Mn_ concentration and have no obvious effect on increasing NH_3_-N concentration. Similarly, results also show that population (pop) has a negative effect both on COD_Mn_ and NH_3_-N, while precipitation (pre) has a negative effect on COD_Mn_ while having no obvious effect on NH_3_-N. Estimated coefficient λ is significantly positive in both Table 4 and Table 5 indicating that the spatial factor has a significant effect on the concentration of these two pollutants.

In summary, the per capita GDP of the Xiangjiang river basin, especially the primary industry output value and industrial output value, population density, and precipitation have different effects on the concentration of pollutants in the basin, especially the impact on COD_Mn_. The reason might be that the Xiangjiang river basin is an economic region in which the first and second industries are the mainstay and advantageous. It is known as the “hometown of nonferrous metals” in which the heavy chemical industry, steel, nonferrous metals, chemicals, and building materials are at the top of the industrial structure. Economic and industrial development have caused serious heavy-metal pollution in Xiangjiang, especially COD_Mn_. Ammonia-nitrogen pollution is mainly caused by domestic wastewater discharged from the cross-strait population and agricultural nonpoint source pollution. With the development of the first industry on the coast, the use of chemical fertilizers and pesticides has been increasing year by year, while the discharge of domestic sewage has also increased.

Research results of Lesage and Pace [54], and Elhorst [55] show that the estimation coefficient of models cannot directly reflect the marginal effect of the independent variable on the dependent variables. The direct and indirect effects of each explanatory variable are listed in Table 6. As is described in the table, the direct effect on COD_Mn_, the per capita GDP (pergdp, pergdp2, pergdp3), primary industry (primary), industry (indus), and population (pop) are significant. The primary industry (primary) in particular has the maximum effect (−6.7732). Additionally, the population (pop) of NH_3_-N has the maximum effect (−4.841386), while precipitation (pre) has the minimum effect (−0.000111).

For the direct effect on COD_Mn_ and NH_3_-N, the per capita GDP was rejected at a 5% significance level and 10% significance level, respectively, which indicates that the per capita GDP has a large spillover effect on the water quality of adjacent areas.

The main reason is that water pollution in the Xiangjiang river basin is serious, and water pollution presents a cross-regional feature. Water pollution in different regions of the basin has spatial correlation. Specifically, when per capita GDP increases in a certain region, water pollution in the adjacent areas is aggravated. The impact of the primary industry on COD_Mn_ and NH_3_-N is not significant, which indicates that the increase in the output value of the primary industry in one region has no effect on the water quality of the adjacent regions. In addition, the impact of industrial and population density on COD_Mn_ is not significant, but that on NH_3_-N is significant, which implies that industrial output value and population density has a positive impact on NH_3_-N pollution in the adjacent areas but has no effect on COD_Mn_.

In summary, in the process of spatial interaction, the space effect is basically reflected in the form of direct effect, while the spatial feedback effect is relatively weak. However, we cannot ignore the indirect influence of the spatial spillover effect of economic development on water pollution.

### 4.3. Intermediate Effect of Industrial Structure

The existing literature shows that there are three factors that determine the shape of the environmental Kuznets curve: scale efficiency, structural effect, and technical effect. In the case where other effects are fixed, the scale effect refers to the concentration of pollutants increasing according to the scale of economic activities; the structural effect refers to the decrease of pollutant concentration when economic activities become clean and green; and the technical effect is the reduced concentration of pollutants when clean technology is used in economic activities. The key points in this section is the structural effect, that is, whether the industrial structure adjustment can change the shape of the EKC curve.

According to Judd and Kenny [56], Baron and Kenny [57], MacKinnon et al. [58], and James Kroes et al. [59], this part carries out a series of tests to verify whether industrial structure has a mediating effect in adjusting the relationship between economic growth and environmental changes. The results show that: (1) when the impact of the economy alone on the environment was considered, the per capita GDP was significantly correlated with COD_Mn_ and NH_3_-N, which indicates that economic growth had a certain influence on the environment (as shown in Table 7); (2) per capita GDP is significantly correlated with the primary industry (Primary) and industry (Indus), implying that economic growth had a certain influence on the development of primary and industries (see Table 8); (3) when considering economic development and the impact of an industrial structure on the environment, the industrial structure has significant impact on COD_Mn_ (see Table 4). Therefore, it can be concluded that the industrial structure has a mediating effect on adjusting the relationship between economic growth and COD_Mn_ concentration change. The impact of an industrial structure on NH_3_-N concentration, however, is not significant (see Table 5), which shows that there is at least one nonsignificant situation. Therefore, the Sobel test was continued, in which test statistic *z* was −0.4228 and 0.3312, respectively, which meant it failed to pass the tests. We can then conclude that the industrial structure is not significant to adjusting the relationship between economic growth and the NH_3_-N concentration change.

In our models, per capita GDP was nonlinearly related to industrial structure and environmental quality; so it was no longer possible to talk about the indirect effect of per capita GDP on environmental quality through the industrial structure as a single quantity. As Hayes et al. [60] describe, we used the rate at which a change in pergdp changes COD_Mn_ concentration indirectly through changes in the industrial structure, denoted as θ, to measure this instantaneous indirect effect. Based on previous estimates, the instantaneous indirect effect of pergdp, corresponding to primary and indus, respectively, is as follows:

(5) $$\begin{array}{ll} \theta_{primary} & =\left(\frac{\partial primary}{\partial pergdp}\right)\left(\frac{\partial CODMn}{\partial primary}\right)\\ & =(-0.0716853+2\times 0.0102196\times \mathrm{pergdp}+3\times (-0.0004045)\times {\left(\mathrm{pergdp}\right)}^{2})\times (-6.7732)\\ & =0.4855-0.1384\mathrm{pergdp}+0.0082{\left(\mathrm{pergdp}\right)}^{2}\\ \theta_{indus} & =\left(\frac{\partial indus}{\partial pergdp}\right)\left(\frac{\partial CODMn}{\partial indus}\right)\\ & =(0.0516433+2\times (-0.0077573)\times \mathrm{pergdp}+3\times (0.0002928)\times {\left(\mathrm{pergdp}\right)}^{2})\times (-2.41538)\\ & =-0.1247+0.0375\mathrm{pergdp}-0.0021{\left(\mathrm{pergdp}\right)}^{2} \end{array}$$

**Figure 4:**
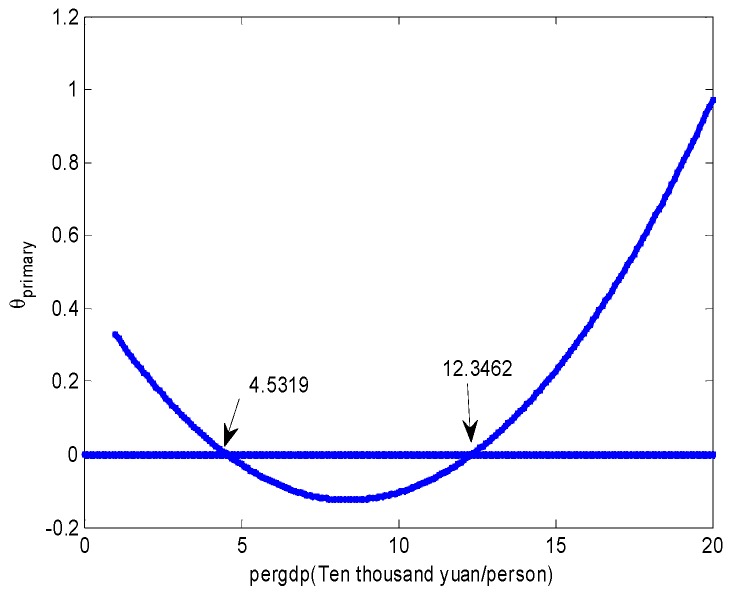
The instantaneous indirect effect of pergdp on COD_Mn_ concentration through primary.

**Figure 5:**
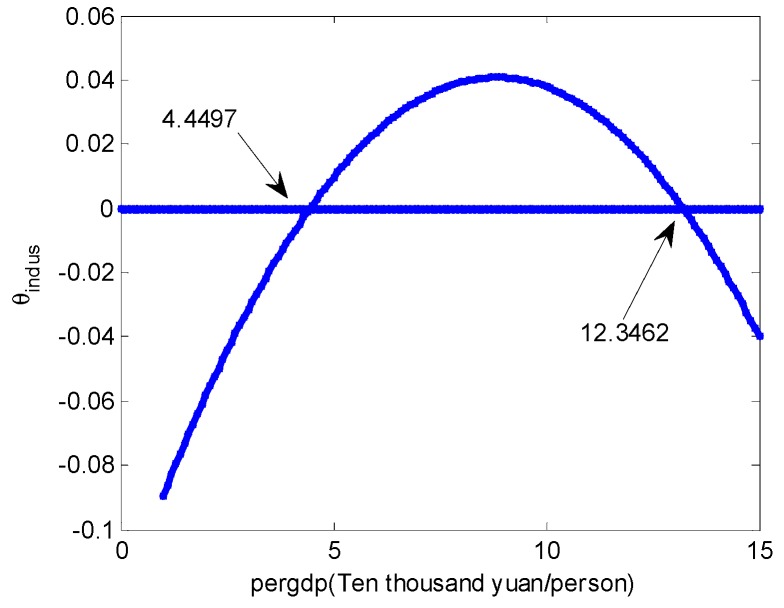
The instantaneous indirect effect of pergdp on CODMn concentration through indus.

Figure 4 graphically depicts the U-shape relationship between pergdp and $\theta_{primary}$. Observe that, when pergdp is between 45,319 and 123,462, the instantaneous indirect effect is negative, meaning that, as pergdp increases, COD_Mn_ concentration decreases through pergdp’s effect on the primary. To the contrary, as pergdp increases, COD_Mn_ concentration increases through pergdp’s effect on indus when pergdp is between 44,497 and 123,462 (Figure 5).

## 5. Conclusions and Policy Implications

This paper examined the COD_Mn_ and NH_3_-N EKC hypothesis using a spatial Durbin model to avoid coefficient-estimation deviation covering the period of 2005–2015 of the Xiangjiang river basin. The results showed that: (1) there exists spatial correlation between COD_Mn_/NH_3_-N and the per capita GDP has a significant influence on COD_Mn_ / NH_3_-N; (2) the turning points of the COD_Mn_ and NH_3_-N environmental Kuznets curves were (83,001, 108,583) and (50,980, 188,931), respectively; (3) the industrial structure can explain the change of the EKC of COD_Mn_, but cannot explain the EKC of NH_3_-N, which indicates that the industrial structure has a mediating effect on CODMn but not on NH_3_-N. Compared with conventional estimation techniques, the spatial econometric approach is seldom used in the validation of a COD_Mn_ / NH_3_-N environmental Kuznets curve. This paper proves that taking spatial autocorrelation into consideration, the spatial panel model is a more proper method, which proposes a more reliable result.

In view of the conclusions above, we make the following policy recommendations to solve environmental problems brought about by economic growth: (1) Spatial measurement results demonstrate that there is an inverted N-shaped relationship between environmental quality and economic development, which indicates a positive interaction between them. Therefore, local government should attach great importance to the inevitability that economic development brings pressure to the environment, and should not simply pursue GDP growth at the expense of environmental pollution. The relationship between economic development and environmental protection should be better dealt with. (2) The reality that upstream and downstream pollution emissions have a strong positive spatial correlation indicates that local government may choose a way to achieve economic development by sacrificing the environmental quality of the surrounding areas. Therefore, it is necessary to stop shortsighted behavior and promote overall environmental quality and consider spatial correlation in the environmental planning of each region. For local government, inter-regional environmental cooperation is of great necessity in dealing with the contradiction between economic development and environmental pollution. Additionally, the national government should pay more attention to the spatial correlation of environmental pollution in the basin so as to implement policies coordinating regional economic development and ecological protection. (3) As the impact of an industrial structure on different pollutants is different, the control of pollutant discharges should be strengthened in a targeted manner during the economic development. Moreover, the reduction effects of different pollutant emissions should be taken into consideration when formulating industrial policies.

## Figures and Tables

**Table 1:** Data statistics for water-quality pollutant concentration in 2005–2015 in the Xiangjiang River.

Variables	Unit	Sample Size	Mean	Maximum	Minimun	Std.Dev
COD_Mn_	mg/L	286	2.706307	8.27	0.25	1.112028
NH_3_-N	mg/L	286	0.5549968	4.17	0.028	0.6375492
Pergdp	10,000 yuan/person	286	3.230058	15.19642	0.5712453	2.394778
primary	%	286	0.132208	0.387262	0.000218	0.104902
indus	%	286	0.398297	0.833231	0.075346	0.156605
pop	10,000 people/km^2^	286	0.134713	1.268224	0.009488	0.222809
ur	%	286	59.85635	100	18.51	26.1837
precipitation	millimeter	286	1368.753	2151	804	253.0147

**Table 2:** Correlation coefficient matrix and Variance Inflation Factor (VIF) test.

Variables	VIF	COD_Mn_	NH_3_-N	Pergdp	Primary	Indus	Pop	Precipitation
COD_Mn_	1.07	1						
NH_3_-N	1.77	0.58 ***	1					
Pergdp	4.93	0.11 *	0.44 ***	1				
Primary	2.71	−0.06	−0.32 ***	−0.74 ***	1			
Indus	1.53	−0.21 ***	−0.38 ***	−0.06	−0.22 ***	1		
Pop	3.64	0.28 ***	0.58 ***	0.79 ***	−0.52 **	−0.35 ***	1	
Precipitation	1.05	−0.19 ***	−0.07	0.16 ***	−0.12 **	0.08	0.06	1

Note: The symbol * denotes *p* < 0.1, ** denotes *p* < 0.05, and *** denotes *p* < 0.01.

**Table 3:** Moran’s I values for COD_Mn_ and NH_3_-N between 2005 and 2015.

Variables	2005	2006	2007	2008	2009	2010	2011	2012	2013	2014	2015
COD_Mn_	0.454	0.336	0.115	0.178	0.305	0.062	0.068	0.193	0.169	0.258	0.408
NH_3_-N	0.647	0.573	0.565	0.569	0.633	0.639	0.338	0.567	0.355	0.632	0.871

*Note*: All the Moran’s I statistics were calculated at a 99% confidence interval.

**Table 4:** Estimation results for COD_Mn_.

Variables	COD_Mn_				
	OLS	SAR	SEM	SDM	
				X	X × W
Pergdp	−0.769125 ** (−2.33)	−0.814879 *** (−2.67)	−0.8491968 *** (−2.78)	−0.8627394 *** (−2.56)	1.586722 *** (3.07)
Pergdp2	0.0568525 (1.43)	0.0632209 * (1.72)	0.0629587 * (1.72)	0.0906988 ** (2.13)	−0.1469894 ** (−2.18)
Pergdp3	−0.0017309 (−1.13)	−0.0019929 (−1.40)	−0.0018965 (−1.36)	−0.0031909 ** (−1.96)	0.0047774 * (1.73)
primary	−7.307696 *** (−3.34)	−7.089124 *** (−3.52)	−7.416741 ** (3.66)	−6.726415 *** (−3.43)	−4.344041 (−1.49)
indus	−2.161912 * (−1.86)	−1.775304 (−1.61)	−1.842487 * (1.69)	−2.326853 ** (−2.07)	−5.136943 *** (−2.56)
pop	−2.429998 (−1.38)	−2.338575 (−1.45)	−2.133634 (−1.31)	−2.405921 (−1.45)	0.3432917 (−0.12)
pre	−0.0002118 (−0.90)	−0.0002282 (−1.06)	−0.0001922 (−0.85)	−0.0000604 ** (−2.11)	−0.0001505 (0.26)
λ		0.095021 (1.40)	0.1135916 (1.52)	0.1003935 * (1.91)	
Log likelihood	−237.9421	−237.2963	−236.7877	−222.8622	
Lagrange Multiplier (LM) spatial lag	3.5722 *				
LM spatial error	4.3314 **				
Robust LM spatial lag	0.2122				
Robust LM spatial error	0.8078				
Durbin–Watson	1.6843	1.7390	1.6887		1.7900
Wald Test		30.2044 ***	30.8764 ***		
LR Test		28.8703 ***	27.8688 ***		
Hausman test				27.9928 **	
Spatial fixed effects	YES	YES	YES	YES	
Time fixed effects	YES	YES	YES	YES	
*R* ^2^	0.0563	0.0604	0.0445	0.153	

Note: The symbol * denotes *p* < 0.1. ** denotes *p* < 0.05. *** denotes *p* < 0.01.

**Table 5:** Estimation results for NH_3_-N.

Variables	NH_3_-N				
	OLS	SAR	SEM	SDM	
				X	X × W
Pergdp	−0.2304252 * (−1.75)	−0.3599694 *** (−3.06)	−0.2796854 ** (−2.40)	−0.3831458 *** (−2.90)	0.5121585 ** (−2.52)
Pergdp2	0.0225424 (1.42)	0.0395953 *** (2.79)	0.0279102 * (1.95)	0.04656 *** (2.83)	−0.0602376 ** (−2.26)
Pergdp3	−0.0002884 * (−0.44)	−0.0010476 * (−1.90)	−0.0006139 (−1.14)	−0.0012895 ** (−2.02)	0.0022042 ** (−2.02)
primary	0.8327864 (0.90)	1.190792 (1.53)	0.7400716 (0.91)	1.028606 (1.33)	−2.10261 * (−1.87)
indus	0.0973589 (0.20)	0.4293576 (1.04)	0.121705 (0.29)	0.6328895 (1.46)	0.1612411 (0.21)
pop	−5.441929 *** (−7.28)	−5.197749 *** (−8.32)	−4.009296 *** (−6.22)	−4.974618 *** (−7.59)	1.229716 (−1.12)
pre	−0.0002017 ** (−2.02)	−0.0001833 ** (−2.20)	0.0002131 ** (−2.16)	−0.0002387 (−1.10)	0.0001564 (0.67)
λ		0.5099835 *** (7.96)	0.474194 *** (5.82)	0.4745402 *** (7.34) 44.0426	
Log-likelihood	7.1233	34.2731	21.5694		
LM spatial lag	32.1044 ***				
LM spatial error	18.5332 ***				
Robust LM spatial lag	29.1668 ***				
Robust LM spatial error	6.6632 **				
Durbin-Watson	1.5847	1.6830	1.6677		1.6600
Wald Test		20.0846 ***	27.0840 **		
LR Test		19.3777 ***	44.6156 ***		
Hausman test				49.4105 ***	
Spatial fixed effects	YES	YES	YES	YES	
Time fixed effects	YES	YES	YES	YES	
*R* ^2^	0.3184	0.1869	0.1911	0.2286	

*Note:* The symbol * denotes *p* < 0.1. ** denotes *p* < 0.05. *** denotes *p* < 0.01.

**Table 6:** Direct and indirect effects of each explanatory variable.

Variables	COD_Mn_			NH_3_-N		
	Direct Effect	Indirect Effect	Total Effect	Direct Effect	Indirect Effect	Total Effect
pergdp	−0.81844 ** (−2.39)	1.6124 *** (3.06)	0.79401 (1.19)	−0.3247845 ** (−2.36)	0.518729 ** (1.88)	0.19339 (0.54)
pergdp2	0.08473 ** (1.97)	−0.14809 ** (−2.14)	−0.0633 (−0.73)	0.0395103 *** (2.28)	−0.06006 * (−1.65)	−0.02055 (−0.44)
pergdp3	−0.002987 * (−1.79)	0.004793 * (1.68)	0.00118 (0.49)	−0.0012895 ** (−1.49)	0.0023856 (−1.58)	0.001367 (0.70)
primary	−6.7732 *** (−3.57)	−5.2055 * (−1.76)	−11.988 *** (−3.33)	0.8134952 (1.08)	−2.45148 (−1.61)	−1.637988 (−0.85)
indus	−2.41538 ** (−2.22)	−5.4209 *** (−2.57)	−7.8363 *** (−3.20)	0.6585608 (1.52)	0.56817 (0.51)	1.2267 (0.91)
pop	−2.4034 (−1.69)	−0.56609 (−0.19)	−2.9695 (−0.78)	−4.841386 *** (−6.93)	−0.58244 (−0.37)	−5.4238 *** (−2.67)
pre	−0.000578 (−0.10)	0.0001696 (0.29)	−0.0001118 (0.44)	−0.0002219 * (−1.88)	0.000111 (0.64)	−0.000111 (−0.85)

Note: The symbol * denotes *p* < 0.1, ** denotes *p* < 0.05, and *** denotes *p* < 0.01.

**Table 7:** Impact of the economy on the environment.

Variables	COD_Mn_	NH_3_-N
pergdp	−0.6344118 *** (−2.59)	−0.4388021 *** (−3.64)
pergdp2	0.0404943 * (1.831)	0.0547024 *** (3.71)
pergdp3	−0.009982 * (−1.89)	−0.00160061 *** (−2.82)
Pop	−2.542371 (−1.47)	−5.144074 *** (−7.82)
pre	−0.0002308 (−0.25)	−0.0001765 (−0.83)
λ	0.171587 *** (2.77)	0.461027 *** (5.76)
Spatial fixed effects	Yes	Yes
Time fixed effects	Yes	Yes
Log-likelihood	−234.0258	40.6721
R^2^	0.0803	0.1857

Note: The symbol * denotes *p* < 0.1, *** denotes *p* < 0.01.

**Table 8:** Impact of the economy on the industry.

Variables	Primary	Indus
pergdp	−0.0716853 *** (−7.8)	0.0516433 *** (3.02)
pergdp2	0.0102196 *** (9)	−0.0077573 *** (−3.77)
pergdp3	−0.0004045 *** (−9.21)	0.0002928 *** (3.69)
Pop	−0.03464 (0.68)	−0.0969328 (−1.07)
pre	−8.14 × 10^−6^ (0.5)	0.0000677 ** (2.30)
λ	0.1769211 * (1.67)	0.1155647 (0.64)
Spatial fixed effects	YES	YES
Time fixed effects	YES	YES
Log-likelihood	763.3026	600.1801
R^2^	0.6333	0.4397

Note: The symbol * denotes *p* < 0.1, ** denotes *p* < 0.05, *** denotes *p* < 0.01.
